# Estimating the potential impact of Attractive Targeted Sugar Baits (ATSBs) as a new vector control tool for *Plasmodium falciparum* malaria

**DOI:** 10.1186/s12936-021-03684-4

**Published:** 2021-03-17

**Authors:** Keith J. Fraser, Lazaro Mwandigha, Sekou F. Traore, Mohamed M. Traore, Seydou Doumbia, Amy Junnila, Edita Revay, John C. Beier, John M. Marshall, Azra C. Ghani, Gunter Müller

**Affiliations:** 1 MRC Centre for Global Infectious Disease Analysis, Department of Infectious Disease Epidemiology, Imperial College London, London, UK; 2 Nuffield Department of Primary Care Health Science, University of Oxford, Oxford, UK; 3 Malaria Research and Training Centre, Faculty of Medicine, Pharmacy and Odonto-Stomatology, University of Sciences, Techniques and Technology of Bamako, BP Bamako, Mali; 4 Department of Public Health Sciences, Miller School of Medicine, University of Miami, Miami, FL USA; 5 Division of Biostatistics and Epidemiology, School of Public Health, University of California, Berkeley, CA USA

**Keywords:** Malaria, Mosquito, Vector control

## Abstract

**Background:**

Attractive targeted sugar baits (ATSBs) are a promising new tool for malaria control as they can target outdoor-feeding mosquito populations, in contrast to current vector control tools which predominantly target indoor-feeding mosquitoes.

**Methods:**

It was sought to estimate the potential impact of these new tools on *Plasmodium falciparum* malaria prevalence in African settings by combining data from a recent entomological field trial of ATSBs undertaken in Mali with mathematical models of malaria transmission. The key parameter determining impact on the mosquito population is the excess mortality due to ATSBs, which is estimated from the observed reduction in mosquito catch numbers. A mathematical model capturing the life cycle of *P. falciparum* malaria in mosquitoes and humans and incorporating the excess mortality was used to estimate the potential epidemiological effect of ATSBs.

**Results:**

The entomological study showed a significant reduction of ~ 57% (95% CI 33–72%) in mosquito catch numbers, and a larger reduction of ~ 89% (95% CI 75–100%) in the entomological inoculation rate due to the fact that, in the presence of ATSBs, most mosquitoes do not live long enough to transmit malaria. The excess mortality due to ATSBs was estimated to be lower (mean 0.09 per mosquito per day, seasonal range 0.07–0.11 per day) than the bait feeding rate obtained from one-day staining tests (mean 0.34 per mosquito per day, seasonal range 0.28–0.38 per day).

**Conclusions:**

From epidemiological modelling, it was predicted that ATSBs could result in large reductions (> 30% annually) in prevalence and clinical incidence of malaria, even in regions with an existing high malaria burden. These results suggest that this new tool could provide a promising addition to existing vector control tools and result in significant reductions in malaria burden across a range of malaria-endemic settings.

**Supplementary Information:**

The online version contains supplementary material available at 10.1186/s12936-021-03684-4.

## Background

Nearly half the world’s population is at risk of contracting malaria [1]. Since the year 2000, the prevalence of its most common and dangerous causative parasite, *Plasmodium falciparum*, has more than halved, leading to the prevention of an estimated 500 million clinical cases of malaria between 2000 and 2016 [2]. This progress has been largely attributed to the scaling-up of vector control tools (VCTs), predominantly long-lasting insecticidal nets (LLINs) and indoor residual spraying (IRS), both of which are now used in malaria-endemic regions across the globe [3]. However, there has been growing concern that an increase in resistance among mosquito vectors to the pyrethroid-based insecticides used in LLINs and IRS is hampering further progress [2]. In addition, it has been suggested that in response to the scaling up of LLINs and IRS which target mosquitoes attempting to feed on humans indoors, the mosquito vectors may be modifying their feeding behaviour to outdoor feeding around dawn and dusk, thus reducing the efficacy of LLINs and IRS [4]. This has led to calls for the development of non-pyrethroid-based insecticides [3] as well as the development and adoption of more effective VCTs specifically targeting outdoor biting [5].

Plant sugars are an essential dietary component for female and male mosquitoes, with female mosquitoes combining this with protein obtained from blood meals to metabolize egg development [6]. Targeting this aspect of the mosquito life cycle using attractive targeted sugar baits (ATSB) [7, 8] (also referred to in the literature as attractive toxic sugar baits) has, therefore, been proposed as a potential strategy that may complement LLINs and IRS in suppressing mosquito vector populations [9–11]. ATSBs provide a manufactured sugar-based alternative to plant sugars for female and male mosquitoes with the addition of a toxin that rapidly kills on ingestion or contact. Previous studies have found the effect of ATSBs to be two-fold. First, ATSBs suppress the overall mosquito population by reducing the numbers of female and male mosquitoes available for reproduction. Second, ATSBs diminish the number of mosquitoes living long enough to pass on the malaria parasite, since many are killed before completing the extrinsic incubation period [12–14]—the length of time between a mosquito biting an infectious human and becoming infectious themselves, typically of the order of ~ 10 days [15]. The suppression of the mosquito population (entomological endpoint) due to the use of ATSBs is expected to result in the reduction of malaria prevalence and clinical incidence (epidemiological endpoints). Although several VCTs have confirmed the entomological endpoint (reduction in catch numbers of several mosquito vector species) attributable to ATSBs [10–14], there is currently no empirical data demonstrating a link between the entomological and epidemiological endpoints for this tool.

A previous study [8] by Marshall et al*.* used a mathematical modelling approach, parameterized using data from a previous ATSB entomological field study in Mali [7], to understand the entomological impact of ATSBs in a West African setting. Mosquito catch number reductions of ~ 80% were projected over a timescale of a few weeks during a time period when the mosquito catch numbers at a control location remained approximately constant. One of the key parameters determining the efficacy of ATSBs on entomological endpoints was the bait feeding rate parameter, which describes the probability of a particular mosquito ingesting the bait on a given day. This was estimated to be ~ 0.4–0.5 per day—a rate substantially higher than the baseline mosquito death rate of ~ 0.1 per day in this setting. Given that mosquito lifetime following ATSB consumption was estimated to be just a few hours [8], this implies a very large reduction in average mosquito lifespan in the presence of ATSBs.

Data from the first cluster-randomized entomological study [16] of ATSBs in Africa was combined with mathematical modelling to explore the potential utility of this new tool to reduce *P. falciparum* malaria prevalence and clinical incidence in humans. The cluster-randomized entomological study was undertaken in southern Mali between April 2016 and December 2017, with the efficacy measured through one-day tests using non-toxic stained bait (to estimate the bait feeding rate), monthly mosquito catches in intervention and control villages and monthly estimates of the entomological inoculation rate (EIR)—the number of infective bites received per person per unit time (to estimate onward infectivity to humans). To estimate the subsequent impact on human endpoints, a mathematical model [17–19] of the transmission of *P. falciparum* malaria to incorporate the presence of ATSBs was adapted, with the field study data being used to estimate key parameters for the model. The model was applied across a range of malaria transmission settings capturing different transmission intensity and seasonality to evaluate the potential utility of ATSBs as an additional VCT.

## Methods

### Entomological study of ATSB

A Phase II entomological study (previously reported in this journal [16]) was undertaken in 14 villages in central Mali. The climate in this region is highly seasonal, with high rainfall in the rainy season (peaking in September) and very dry conditions in the dry season (December–March). Full details of the study and outcomes are reported elsewhere and are summarized here for completeness.

Fourteen villages were selected to participate in the study. In the first year of the study (April 2016 to May 2017), baseline entomological data were collected in all 14 villages. They were randomly sorted into two groups of seven, with one group designated as the intervention (ATSBs + standard of care) group and one as the control (standard of care) group. ATSBs were then deployed in the intervention villages in June 2017 with two bait stations containing the insecticide dinotefuran being placed on the outer walls of each building, and entomological data collected through to December 2017. To estimate the feeding rate on the ATSBs, 1-day tests using stained bait were carried out at monthly intervals in the control villages. As a means of estimating the bait-feeding rate, simple tests were carried out in which attractive sugar baits without toxic additives (ASBs) were temporarily introduced to villages where ATSBs were not used. These baits contained a harmless dye which allowed captured mosquitoes in the relevant villages to be separated into those which had fed on the ASBs and those which had not.

Mosquitoes were collected monthly in each village using Centre for Disease Control (CDC) UV light traps, Malaise traps and pyrethroid spray catch (PSC) inside houses. Here the data from CDC traps is used as a measure of mosquito density. In addition, human landing catch (HLC) measurements were carried out indoors and outdoors by four volunteers (two indoors in separate homes and two outdoors at least 5 m apart from the indoor volunteers). A random sample of the captured mosquitoes were examined to determine the proportion containing viable sporozoites and, therefore, onwardly infectious; this sporozoite rate is multiplied by the number caught per volunteer per unit time in HLC experiments to estimate the entomological inoculation rate (EIR).

### Estimating the impact of ATSBs on mosquito density and EIR

To estimate the impact of the ATSBs on the two entomological endpoints—mosquito count and EIR—a non-linear model was formulated to capture the seasonal variation in outcomes in addition to the effect of the intervention whilst accounting for the village cluster-level variability. The non-linear model with mosquito count outcomes from the CDC light traps had convergence difficulties. *M*_*i*_ denotes the mosquito population count in village *i* where *i* = *1, 2…14* (7 treatment and 7 control villages) at time *t* [indexing the month of the year in which count of mosquito outcome was obtained with July being the first month (t = 1) and November the fifth month (t = 5)].

Equation 1a shows the trigonometric function, which captures the seasonal variation in the mosquito population density for an individual village where *a*_*M*_*, b*_*M*_*, c*_*M*_*, d*_*M*_ are parameters to be estimated. The term *R*_*M*_ denotes the treatment effect coefficient for ATSB (the fractional decrease in population density) while *δ*_*T*_ is the predictor that identifies treatment assignment at village level (coded as 1 and 0 for treatment and control villages respectively). The variation in count between villages was captured by a Poisson distribution with its mean based on Eq. 1a (Eq. 1b).

1a $$M\left( {\text{t}} \right) = \left( {1 - {R_M}{\delta_T}} \right)({{\text{a}}_M}\sin \left( {{{\text{b}}_M}{\text{t}} - {{\text{c}}_M}} \right) + {{\text{d}}_M})$$

1b $${{\text{M}}_{\text{i}}}\left( {\text{t}} \right)\ \sim{\text{Poi}}({\uplambda } = {{\text{e}}^{\left( {{\text{r}}{_{{\text{M}},{\text{i}}}} + \left( {1 - {{\upbeta }_M}{{\updelta }_{\text{T}}}} \right)} \right)}} \cdot \left( {{{\text{a}}_M}\sin \left( {{{\text{b}}_M}{\text{t}} - {{\text{c}}_M}} \right) + {{\text{d}}_M})} \right)$$

The terms *r*_*M,i*_ denotes the random intercept that captures the correlation of mosquito population count at village level assumed to be be normally distributed as *r*_*M,i*_ ~ *N(0,σ*_*M,r*_^*2*^*)* where *σ*_*M,r*_^*2*^ is a level 2 variance component to be estimated. The reduction *R*_*M*_ in mosquito count can be approximated by the formula ${R}_{M}\sim \left(\mathrm{exp}\left(1\right)-\mathrm{exp}(1-{\beta }_{M})\right)/\mathrm{exp}(1)$ where *β*_*M*_ is the effect size parameter estimated from fitting the statistical model from Eq. 1b.

The EIR is modelled by a normal distribution with its parameters captured by the model shown in Eq. 2. The mean of the normal distribution was defined by the term $\left(1-{R}_{EIR}{\delta }_{T}\right)({a}_{EIR}\mathrm{sin}\left({b}_{EIR}t-{c}_{EIR}\right)+{d}_{EIR})+ {r}_{EIR,i}$ and the residual error variance by *ε*_*i*_.

2 $$EI{R_i}\left( t \right) = \left( {1 - {R_{EIR}}{\delta_T}} \right)({a_{EIR}}\sin \left( {{b_{EIR}}t - {c_{EIR}}} \right) + {d_{EIR}}) + \;{r_{EIR,i}} + {\varepsilon_i}$$

Due to the nature of the data, the time *t* was measured in months in which the EIR value was obtained. The months were coded as t = 1 to 7 representing the months from June to December. As with Eq. (1), the fixed effect part of Eq. 2 containing the term *a*_*EIR*_* sin(b*_*EIR*_*t – c*_*EIR*_*)* + *d*_*EIR*_ is a trigonometric function that captures the seasonal variation in the EIR where *a*_*EIR*_*, b*_*EIR*_*, c*_*EIR*_*, d*_*EIR*_ are parameters to be estimated. The term *R*_*EIR*_ denotes the treatment effect coefficient for ATSB (the fractional decrease in EIR) while *δ*_*T*_ is the predictor that identifies treatment assignment at village level (coded as 1 and 0 for treatment and control villages, respectively). The terms *r*_*EIR,i*_ and *ε*_*i*_ denote the random intercept that captures the correlation of EIR at village level and the residual variability, respectively. These are assumed to be normally distributed as *r*_*EIR,i*_ ~ *N(0,σ*_*EIR,r*_^*2*^*)* and *ε*_*ij*_ ~ *N(0,σ *_*EIR,e*_^*2*^*)* where *σ *_*EIR,r*_^*2*^ and *σ *_*EIR,e*_^*2*^ are variance components to be estimated.

In both cases, the model was fitted to the mosquito catch and EIR data using PROC NLMIXED with the adaptive Gauss-Hermite quadrature method [20] in Statistical Analysis System (SAS) software version 9.4 [21] to obtain the point estimate for the parameter *R* together with its corresponding 95% confidence interval based on a two-sided p-value for the null hypothesis ${H}_{0} :R=0$ *versus* the alternative${H}_{A} :R\ne 0$. The parameter values extrapolated from population and EIR data are shown in Additional file 1.

### Estimating the excess mortality

Equation 3a expresses the rate of change of the mosquito catch in ATSB villages *M*_*EXP*_ following the introduction of ATSBs. This expression, based on the approach taken by Marshall et al*.* [8], is a simplified version of the more detailed mosquito population model, used here as a means of relating the function fitted to the observed data (Eq. 1) to mosquito mortality parameters.

3a $$\frac{{d{M_{EXP}}\left( t \right)}}{dt} = {\mu_{BASE}}{M_{EQ}}\left( t \right) - \left( {{\mu_{BASE}} + {\mu_{ATSB}}} \right){M_{EXP}}\left( t \right)$$

3b $$\frac{{d{M_{CON}}\left( t \right)}}{dt} = {\mu_{BASE}}{M_{EQ}}\left( t \right) - {\mu_{BASE}}{M_{CON}}\left( t \right)$$

Here *M*_*EQ*_ is the seasonally varying equilibrium mosquito catch, *µ*_*BASE*_ is the baseline adult mosquito appearance and death rate in the absence of ATSBs, and *µ*_*ATSB*_ is the excess mortality due to ATSBs. *µ*_*BASE*_ is given by the natural mosquito death rate *µ*_*NAT*_ added to any additional mortality due to vector control interventions present in both control and ATSB villages. In control villages, the mosquito catch *M*_*CON*_ is given by the same equation with *µ*_*ATSB*_ set to 0 (Eq. 3b).

From Eq. 1a, the average mosquito catch rate in the control and ATSB arms can be written as shown in Eqs. 4a, b.

4a $${M_{CON}}\left( t \right) = {a_M}\;{\text{sin}}\left( {{b_M}t - {c_M}} \right) + {d_M}$$

4b $${M_{EXP}}\left( t \right) = \left( {1 - {R_M}} \right){M_{CON}}\left( t \right) = \left( {1 - R} \right)\left[ {{a_M}\;{\text{sin}}\left( {{b_M}t - {c_M}} \right) + {d_M}} \right]$$

The relationship between *M*_*EXP*_ and *M*_*CON*_ (Eq. 4b) can be substituted into Eq. 3a (Eq. 5a). Equation 5a and Eq. 3b can then re-arranged to give two different expressions for *M*_*EQ*_ (Eqs. 5b–c). These can then be equated in order to express the relationship between *M*_*CON*_, *R*_*M*_, *µ*_*BASE*_ and *µ*_*ATSB*_ (Eq. 5d).

5a $$\left( {1 - {R_M}} \right)\frac{{d{M_{CON}}}}{dt} = {\mu_{BASE}}{M_{EQ}} - \left( {1 - {R_M}} \right)\left( {{\mu_{BASE}} + {\mu_{ATSB}}} \right){M_{CON}}$$

5b $${M_{EQ}} = \frac{{1 - {R_M}}}{{{\mu_{BASE}}}}\left( {\frac{{d{M_{CON}}}}{dt} + \left( {{\mu_{BASE}} + {\mu_{ATSB}}} \right){M_{CON}}} \right)$$

5c $${M_{EQ}} = \frac{1}{{{\mu_{BASE}}}}\frac{{d{M_{CON}}}}{dt} + {M_{CON}}$$

5d $${\mu_{ATSB}} = \frac{{R_M}}{{1 - {R_M}}}\left( {\frac{1}{{{M_{CON}}}}\frac{{d{M_{CON}}}}{dt} + {\mu_{BASE}}} \right)$$

Equation 5d can be rewritten as follows with Eq. 4a used to substitute for *M*_*CON*_, to give an estimate of *µ*_*ATSB*_ in terms of the estimated parameters *a*_*M*_, *b*_*M*_, *c*_*M*_, *d*_*M*_, *R*_*M*_ and the base death rate:

6 $${\mu_{ATSB}} = \frac{{R_M}}{{1 - {R_M}}}\left( {\frac{{{a_M}{b_M}\;{\text{cos}}\left( {{b_M}t - {c_M}} \right)}}{{{a_M}\;{\text{sin}}\left( {{b_M}t - {c_M}} \right) + {d_M}}} + {\mu_{BASE}}} \right)$$

### Estimating the impact of ATSBs on malaria prevalence and incidence

An existing detailed model [17–19, 22] of malaria was used for simulations of the effects of ATSBs on malaria infection levels in human populations. In the model, individuals begin life susceptible to *P. falciparum* infection and are exposed to infectious bites at a rate that depends on local mosquito density and infectivity. Newborn infants passively acquire maternal immunity, which decays in the first 6 months of life. After exposure, individuals are susceptible to clinical disease and may progress through a range of infection categories (clinical infection, asymptomatic infection, subpatent infection, treated and prophylaxis). As they age, the risk of developing disease declines through natural acquisition of immunity, at a rate that depends on the rate of continued exposure. At older ages, parasitaemia levels fall so that a high proportion of asymptomatic infections become sub-microscopic. Full mosquito-population dynamics were included in the model to capture the effects of vector control in preventing transmission, killing adult female mosquitoes, and the resulting reduction in egg-laying. The model has previously been fitted to existing data on the relationship between rainfall (the seasonal parameter found to give best fit to data [22]), mosquito abundance, entomological inoculation rate (the rate at which people receive infectious bites), parasite prevalence and clinical disease incidence in order to establish parameter values. Full mathematical details of the model and a complete parameter list are included in Additional file 1.

The effect of ATSBs was included in the model by modifying the death rate of mosquitoes from *µ*_*BASE*_ to *µ*_*BASE*_ + *µ*_*ATSB*_ as shown in the previous section. Note that this differs from the modelling of other common vector control interventions such as LLINs and IRS, where direct reduction in biting rate must also be incorporated and additional mortality is affected by biting rate [23].

The initial conditions for a study were created by generating characteristics (proportions of humans in different infection categories, immunity levels) at steady state under particular levels of adult mosquito density, then after an extended period of time with particular seasonal variation in adult mosquito density. ATSBs were then introduced to modify the mosquito death rate, resulting in reduced mosquito populations due to direct death and reduced larval birth rate. As noted above, the population of infectious mosquitoes decreases more significantly than the overall population, due to increased death rates causing fewer infected mosquitoes to survive for the duration of the parasite incubation period. This in turn caused reductions in EIR which in turn reduced the number of new infections. Benchmark data values including malaria prevalence and clinical incidence were recorded at regular intervals and the results compared with the same data values under control conditions (where the mosquito death rate is simply equal to the natural value *µ*_*NAT*_) to measure the effectiveness of ATSBs.

## Results

### Impact of ATSBs on mosquito catch numbers and EIR in Malian villages

A cluster-randomized entomological study [16] was undertaken in 14 villages in southern Mali between April 2016 and December 2017. In the first year of the study (April 2016 to May 2017) baseline entomological data were collected in all 14 villages. ATSBs were then deployed in the seven intervention villages in June 2017, and entomological data collected through to December 2017.

**Fig. 1:**
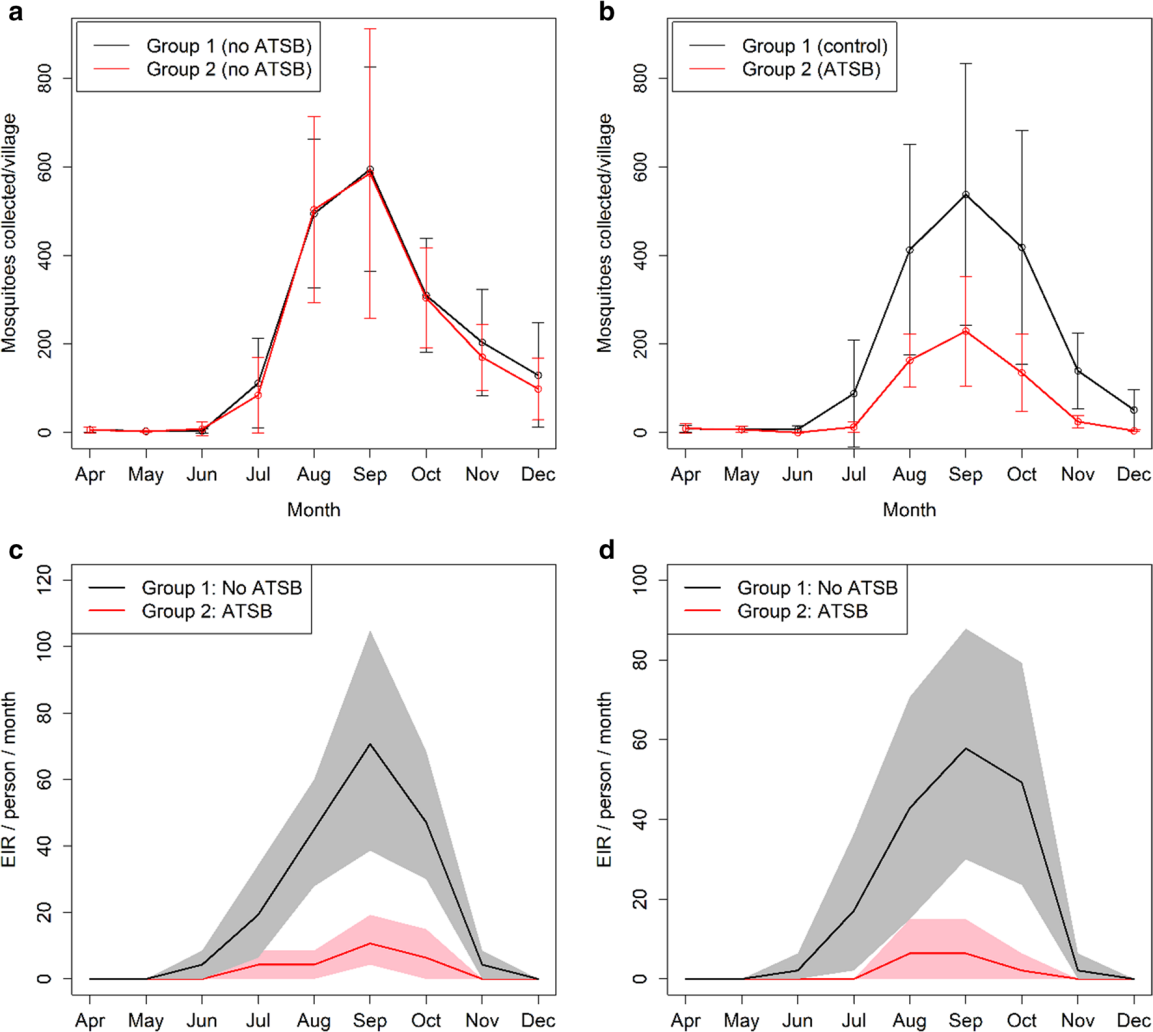
**a**, **b** Number of female mosquitoes caught per village using CDC traps in 2016 (**a**) and 2017 (**b**) for each group of 7 villages [16]. ATSBs were introduced in Group 2 in 2017. Error bars show the standard deviation between villages. **c**, **d** Estimated EIR in ATSB and control villages calculated from the fraction of mosquitoes infected among those caught using the human landing catch method in 2017, split into indoor (**c**) and outdoor (**d**) collection [16]. Shaded regions show 95% bootstrap percentile interval based on 5000 bootstrap samples

Figure 1a, b shows monthly mosquito catch number data (collected using CDC traps) for the two arms of the study from April to December in 2016 and 2017. Whilst there was substantial variation between villages, as illustrated by the error bars, there was no significant difference in the average number of collected mosquitoes per village between the two arms (Groups 1 and 2) during the baseline period (Fig. 1a, average reduction factor *R* = 0.017, *p* = 0.90. Following the intervention with ATSBs, there was a significantly lower mosquito count in the intervention villages compared to the control villages, with a 57% average reduction in mosquito count (Fig. 1b, R = 0.572, 95% confidence interval 33–72%, *p* < 0.001). Figure 1c, d shows the estimated EIR in the intervention and control arms for the five wettest months of 2017 (dry months were excluded due to the small number of mosquitoes caught). A substantially greater reduction in the EIR is obtained: 89% (*R* = 0.890, 95% confidence interval 75–100%, *p* < 0.001) for indoor human landing catch (HLC) and 93% (*R* = 0.9208, 95% confidence interval 75–100%, *p* < 0.001) for outdoor HLC. This indicates that, as anticipated, the effect of ATSBs on the malaria infection rates is greater than that which can be inferred from reductions in mosquito catch numbers, due to an elevated mosquito death rate reducing the population of older females and hence the average mosquito lifespan. This in turn reduces the number of infected mosquitoes which survive the extrinsic incubation period.

### Estimated bait feeding and killing rates

**Fig. 2:**
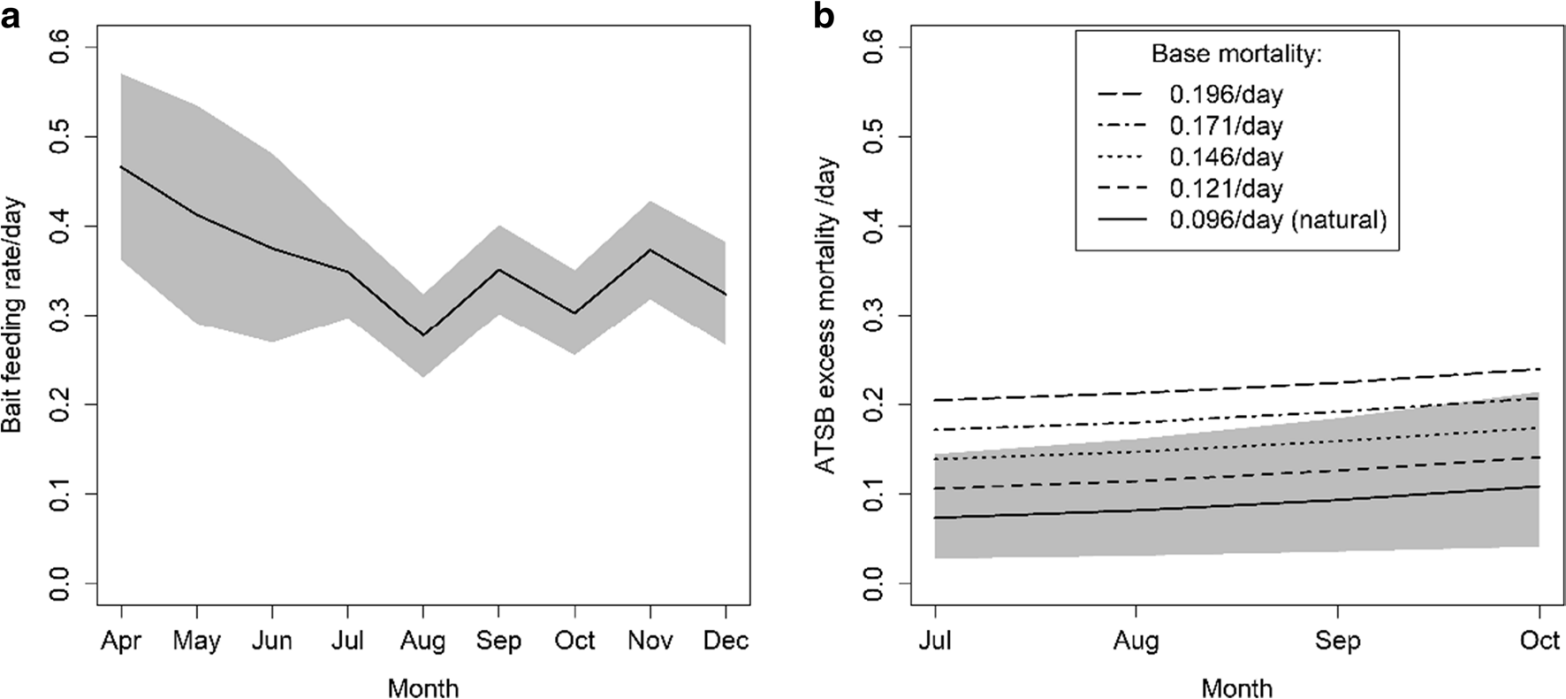
**a** Estimated bait feeding rate in control villages, calculated as the fraction of female mosquitoes stained in 1-day tests using non-toxic but food dye-stained bait between April and December 2017. Shaded region represents binomial confidence interval. **b** Estimated excess mortality rate *µ*_*ATSB*_ in intervention villages, calculated as the additional death rate (see Eq. 2) required to reproduce the observed difference in mosquito numbers between the intervention and control villages (using functions fitted to data as described in Methods). Shaded region represents 95% confidence interval where base mortality = natural mortality rate 0.096/day

Figure 2a shows estimates of the bait feeding rate calculated from 1-day staining tests using non-toxic bait from the 2017 study. The proportion of female mosquitoes stained by the baits 24 h after their introduction ranges from 0.28–0.38 per day during the period when ATSBs were in use (June-December). The proportion of mosquitoes stained is generally highest in the drier months of the year when measurements were taken (April–May) and lowest in the wetter months (August–December). The monthly statistical estimates of the effect size of the intervention on mosquito populations (*R*) from 2017 were used to estimate the excess mortality by transforming a birth–death model for the mosquito population. Figure 2b shows the results. The values vary in the range 0.07–0.11 per day (mean 0.09 per day) when the baseline mortality *µ*_*BASE*_ is 0.096/day (based on the value used for the natural mosquito death rate *µ*_*BASE*_, as shown in the lower line in Fig. 2b. These values are notably lower than the estimated bait feeding rate shown in Fig. 2a.

If the baseline mortality *µ*_*BASE*_ is increased, higher values of *µ*_*ATSB*_ are obtained, as shown in the upper lines in Fig. 2b. This represents additional baseline mortality above the value of *µ*_*NAT*_ present in both control and intervention villages due to non-ATSB vector control interventions. Long-lasting insecticidal nets were present in the study region at high coverage [16], but a figure for the additional baseline mortality cannot be estimated accurately as the efficacy (which can vary depending on usage patterns and insecticide resistance) is not known. The value obtained where *µ*_*BASE*_ = *µ*_*NAT*_ is used in the remainder of this paper as a conservative estimate of the actual excess mortality and an effective value applicable to calculations where LLINs are not incorporated.

**Fig. 3:**
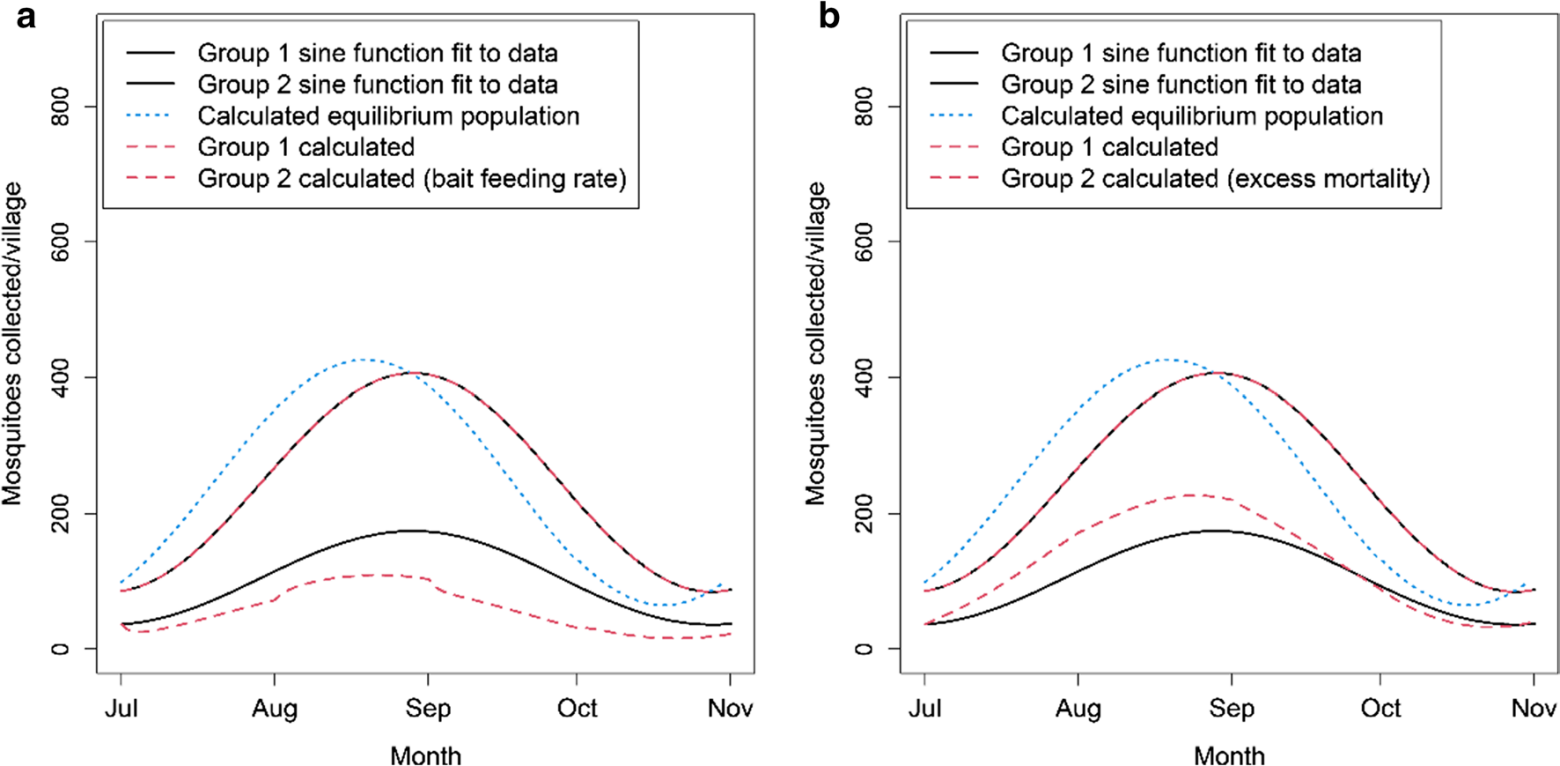
Sine functions fitted to 2017 mosquito collection data for Groups 1–2, compared with values calculated using Eqs. 3a-b, with equilibrium values M_EQ_ calculated using Eq. 5b. Calculated values for Group 2 shown for **a** excess mortality given by 1-day staining tests (Fig. 2a) **b** mean excess mortality extrapolated from fitting functions (Fig. 2b)

Next, the simplified mathematical model of the mosquito population dynamics was used to predict the mosquito catch rate with and without the ATSB intervention, using either the bait feeding rate or the excess mortality as estimates to parameterize the impact of ATSBs. The model outputs based on the bait feeding rates estimated from 1-day staining tests overestimate the observed reduction in the mosquito catch (Fig. 3a). In contrast, using the excess mortality estimated from catch data results in model output which more closely mirrors the observed mosquito counts (Fig. 3b).

### Predicted impact of ATSBs on malaria transmission

**Fig. 4:**
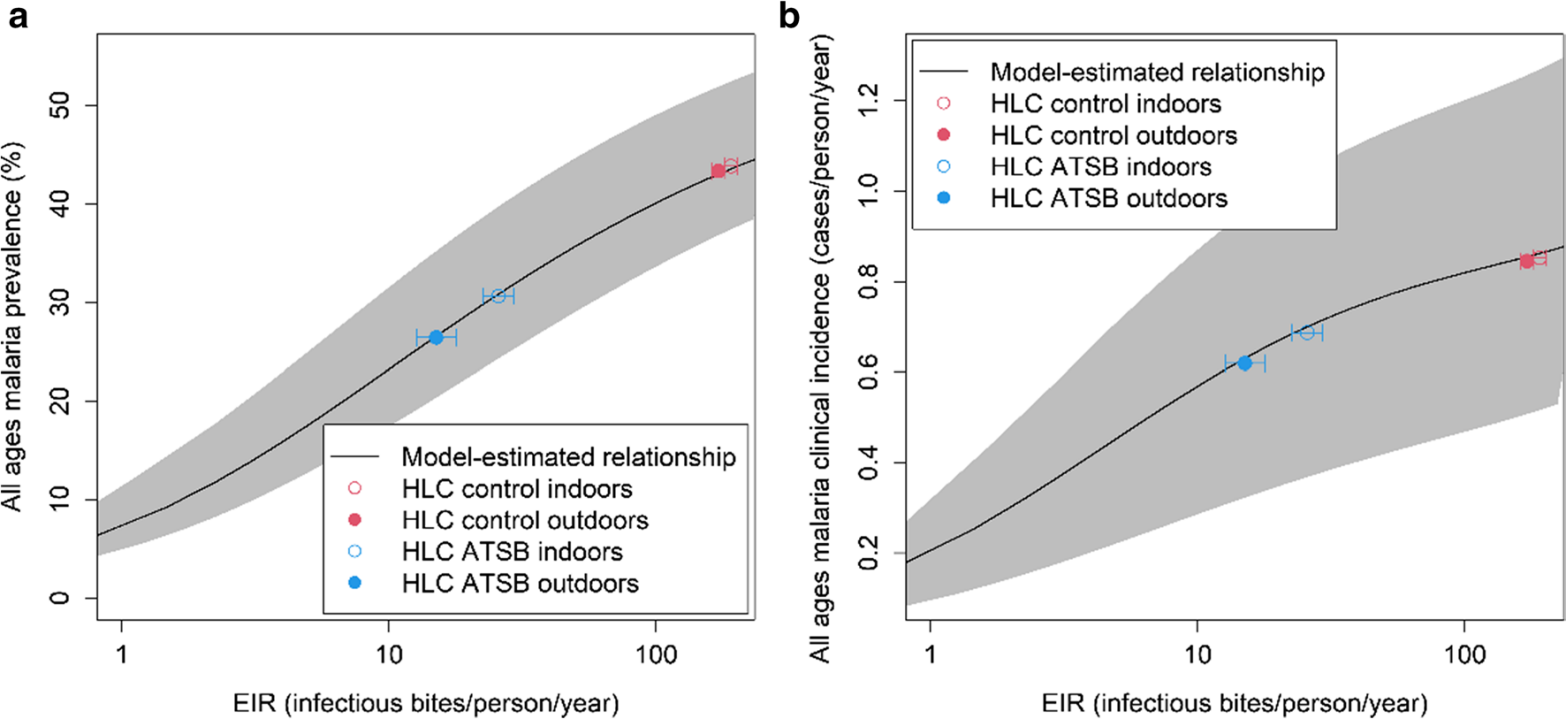
The lines show the equilibrium year-round average model-estimated **a** all-age parasite prevalence and **b** all-age clinical incidence (cases per person per year), plotted against annual entomological inoculation rate (EIR). The coloured points show annual EIR values calculated from field data. The shaded regions correspond to the 95% posterior credible intervals for the modelled relationship between EIR, prevalence and incidence (see Methods)

To obtain preliminary estimates of the impact of ATSBs on human endpoints, the changes in malaria prevalence and incidence in humans expected to be produced by ATSBs were calculated based on the measured impact on EIR in the field study. This was carried out using model-estimated relationships between EIR, parasite prevalence and clinical incidence previously obtained from fitting to data on these three metrics [17–19]. Figure 4 shows the equilibrium relationship (obtained by running the model for 10 years from initial values calculated for steady-state at constant rainfall) between annual EIR and all-ages parasite prevalence (Fig. 4a) or clinical incidence (Fig. 4b) averaged over the year using the seasonal rainfall variation in the study area in Mali. The estimates of EIR from the HLC data (Fig. 3) are shown super-imposed on this profile. From this relationship, the observed reduction in EIR values corresponds to an approximate reduction in all-ages prevalence from 43% (95% CrI 37–52%) to 27% (95% CrI 20–35%) or 44% (95% CrI 37–53%) to 31% (95% CrI 25–40%) for outdoor and indoor HLC collection, respectively, and a reduction in annual all-age clinical incidence from 0.85 (95% CrI 0.51–1.26) or 0.86 (95% CrI 0.52–1.27) cases per person per year to 0.63 (95% CrI 0.33–0.96) or 0.70 (95% CrI 0.37–1.05) cases per person per year.

**Fig. 5:**
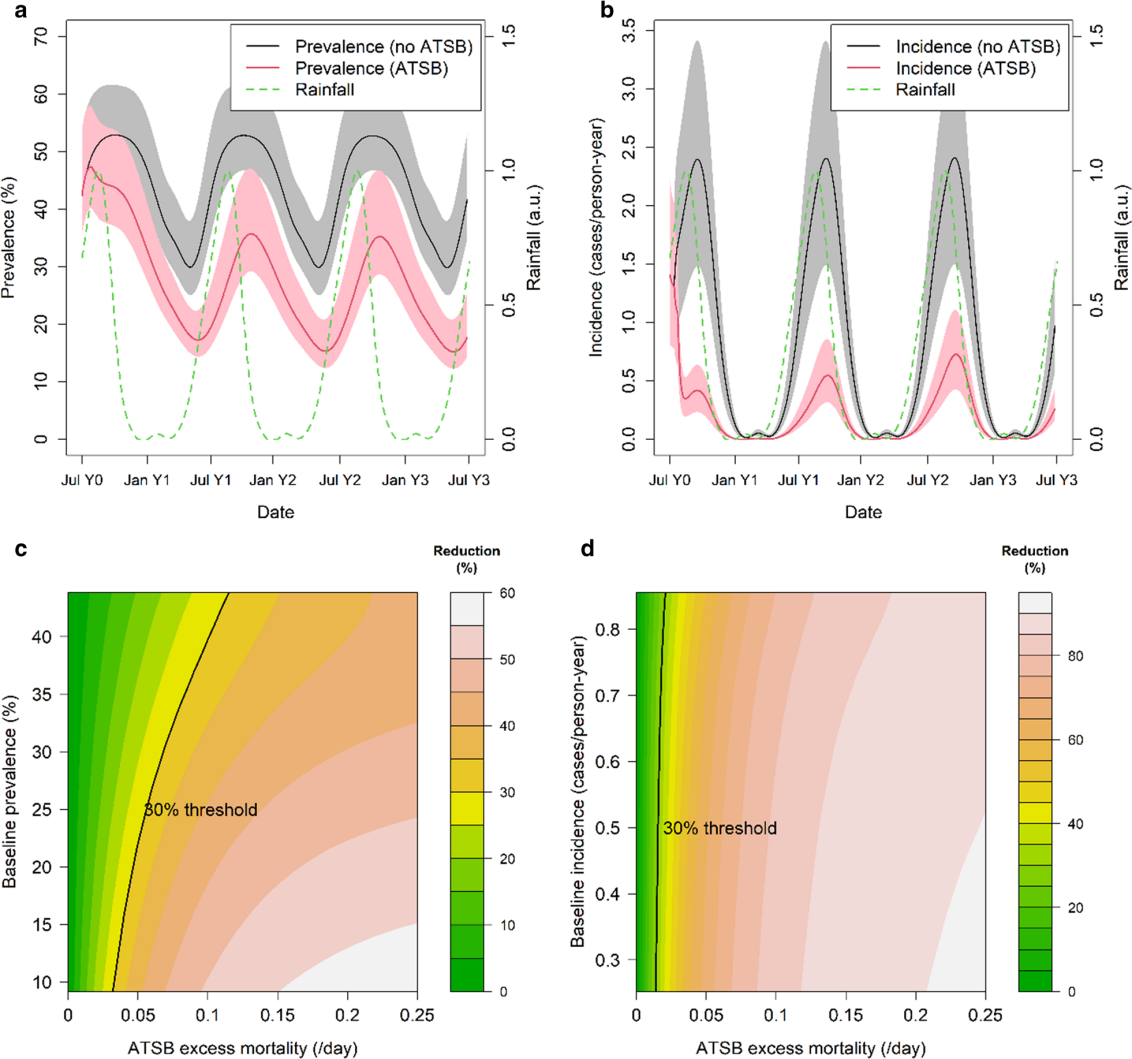
**a**, **b** Model-predicted all-ages parasite prevalence (**a**) and clinical incidence (**b**) over the course of 3 years after introduction of ATSBs (red line) and without ATSBs during the same period (black line). The green dotted line shows the assumed rainfall pattern (in arbitrary units). For these runs the excess mortality *µ*_*ATSB*_ is set to the average value estimated from field trial results (0.09/day). Shaded areas represent range of values obtained using model parameters in 95% credible interval. **c**, **d** Model-predicted reduction in all-age year-round parasite prevalence (**c**) and clinical incidence (**d**) in first year of ATSB use as a function of prevalence/incidence under non-ATSB conditions and ATSB excess mortality µ_ATSB._ All simulations use the seasonal Mali rainfall profile shown in **a** and **b**

To obtain more detailed predictions of the impact of ATSBs across different malaria transmission levels that take into account the dynamics generated by changes in immunity in the human population, outputs were generated from the mathematical model of *P. falciparum* malaria transmission using the estimated excess mortality from the field study and with the seasonality in transmission determined by rainfall patterns in Mali. Figure 5a, b shows predicted changes in parasite prevalence (Fig. 5a) and clinical incidence (Fig. 5b) over the course of 1 year, at a baseline malaria level corresponding to the highest annual EIR measured in the field study (~ 190/person-year). Here the excess mortality is assumed to be constant over the course of the year. In this highly seasonal setting, clinical incidence is predicted to be concentrated during the malaria transmission season, whilst parasite prevalence is predicted to show less seasonal variation. The greatest observable impact of ATSBs is predicted to occur in clinical incidence; however, substantial reductions are also predicted to be observed in parasite prevalence. Furthermore, more substantial reductions in clinical incidence are predicted than were obtained from the equilibrium relationships; this is in part due to the benefit from higher levels of pre-existing immunity which are expected to decay over subsequent years if the intervention is maintained. However, the study also showed a lower impact on the HLC endpoint underlying the EIR estimates compared to the CDC trap data used for our dynamic model projections [16].

Figure 5c, d shows the predicted reductions in parasite prevalence and clinical incidence due to ATSB for a range of excess mortality values (on the x-axis) and baseline transmission levels (on the y-axis). In all settings, the predicted impact is large even for relatively low excess mortality values. Notably, a greater reduction in clinical incidence is predicted compared to the reduction in parasite prevalence in areas with high levels of malaria at baseline. A 30% reduction (highlighted on each graph) is predicted for clinical incidence when the excess mortality is less than 0.05, even in areas with high baseline malaria. For parasite prevalence, the excess mortality required to achieve this threshold varies more strongly with the baseline malaria transmission level, but even when the baseline year-round all-age prevalence is as high as 45%, a reduction of 30% is predicted with an excess mortality above 0.1.

**Fig. 6:**
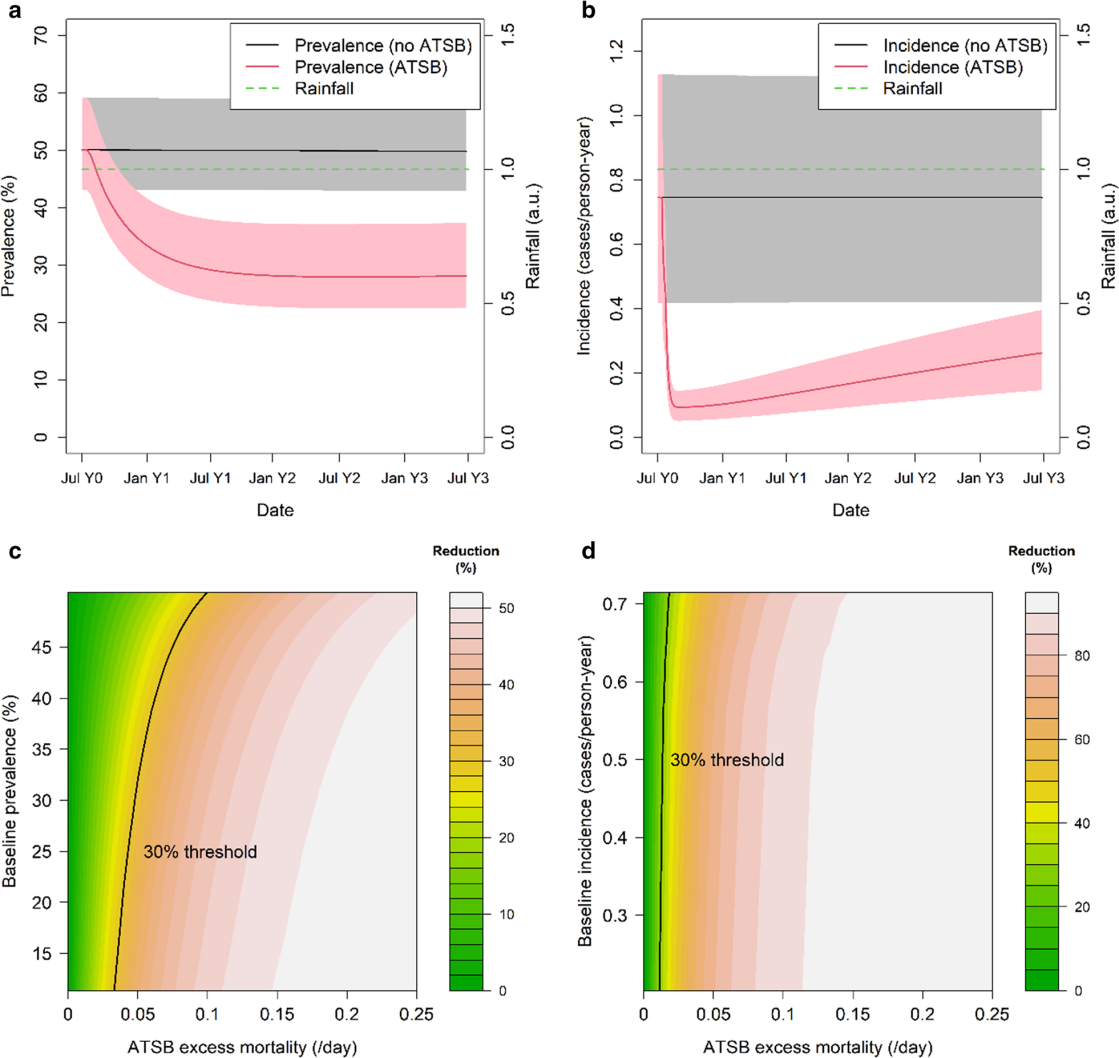
**a**, **b** Model-predicted all-age parasite prevalence (**a**) and clinical incidence (**b**) over the course of 3 years after introduction of ATSBs (red line) and without ATSBs during the same period (black line) under the assumption of constant rainfall (green dotted line) For these runs the excess mortality *µ*_*ATSB*_ is set to the average value estimated from field trial results (0.09/day). Shaded areas represent range of values obtained using model parameters in 95% credible interval. **c**, **d** Model-predicted reduction in all-ages year-round prevalence (**c**) and clinical incidence (**d**) as a function of prevalence/incidence under non-ATSB conditions and ATSB excess mortality µ_ATSB_, assuming constant rainfall

Next, the model was used to understand whether these results would differ in areas without such strong seasonal patterns of malaria. Figure 6 shows the same outputs as Fig. 5 but for constant rainfall as opposed to the highly seasonal rainfall patterns observed in the region in which the field study was conducted. Although the dynamics of the effect vary as expected (Fig. 6a, b), the overall percentage reduction in year-round prevalence and incidence for a given baseline malaria transmission level (Fig. 6c, d) is predicted to be similar to that predicted in areas with seasonally-varying rainfall.

**Fig. 7:**
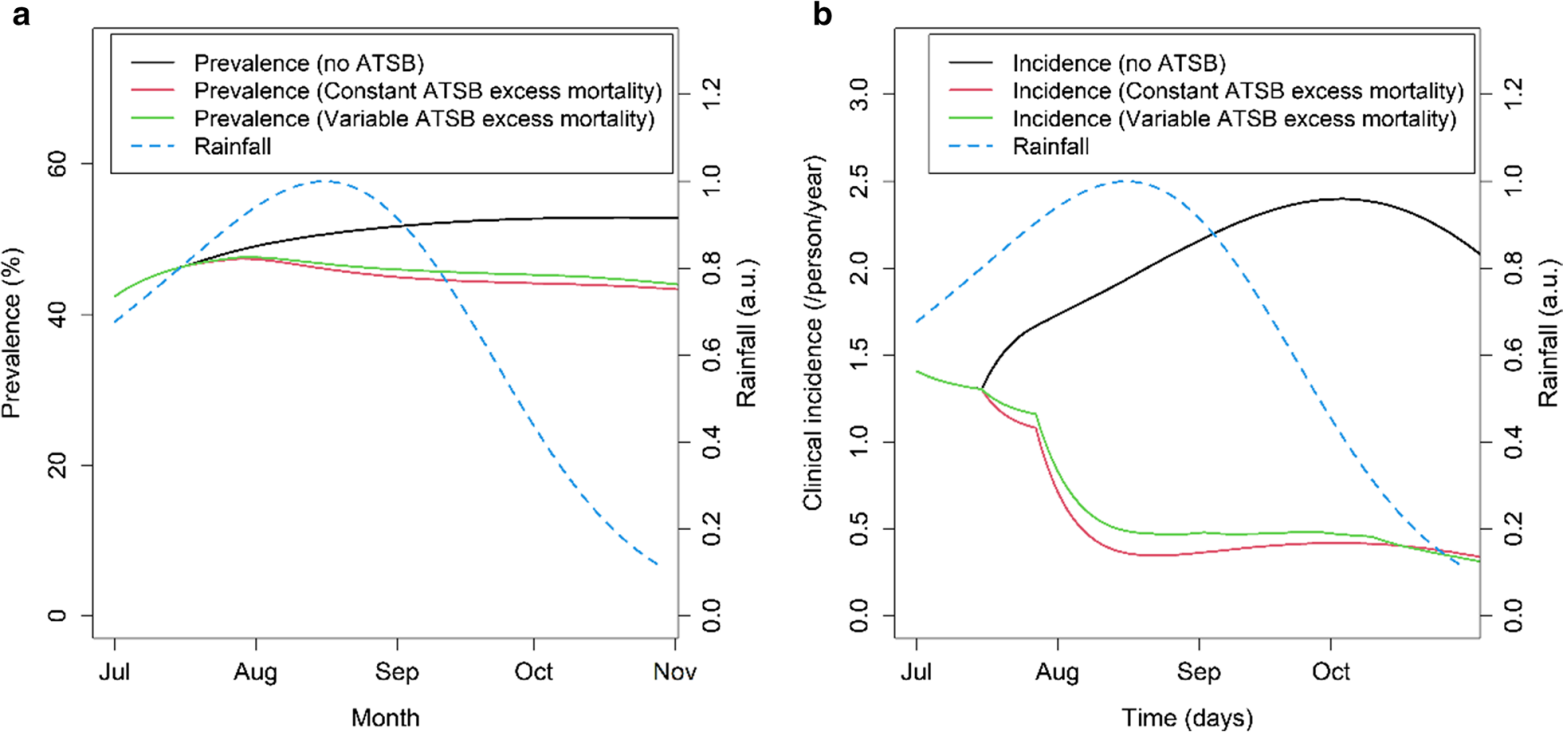
Predicted time progression of all-age parasite prevalence (**a**) and clinical incidence (**b**) under Mali rainfall conditions, over 120-day period representing the time period used to estimate the ATSB excess mortality µ_ATSB_. Values are shown for µ_ATSB_ values of zero (control), for the average value estimated from field trial results (0.09/day), and for the variable values shown in Fig. 2b. Credible intervals are not shown here as the red and green curves overlap

The study showed a seasonally variable excess mortality, with higher rates estimated during the drier months and lower rates during the wettest months. The effect of seasonal variation in the excess mortality on the predicted impact of ATSBs was also explored. Figure 7 shows the predicted parasite prevalence and incidence over time for in the absence of ATSBs, with ATSBs assuming a constant excess mortality and with ATSBs displaying a variable excess mortality based on the results shown in Fig. 2b. Overall, a small (< 10%) reduction in the impact of the intervention is predicted if the ATSB excess mortality varies in the pattern observed in the field study.

## Discussion

The results from the first cluster-randomized entomological field study of ATSBs [16] demonstrate the potential of this new tool to significantly suppress *Anopheles* catch numbers, confirming results from earlier studies [8, 11, 13]. Using this data, a statistically significant reduction in the mosquito count in the villages with ATSBs and LLINs is estimated compared to those with LLINs alone of 57% (95% CI 33–72%) over a 1-year follow-up period. Notably, this effect is most apparent in the reduction in the seasonal peak mosquito catch rate concomitant with the period of highest malaria transmission. Furthermore, a greater estimated reduction in onward transmission as captured by the EIR of 91% (95% CI 75–100%) is obtained, reflecting the impact that this intervention is likely to have in reducing the lifespan of mosquitoes and hence the potential for mosquitoes to survive the extrinsic incubation period. These modelling results suggest that these large reductions in vector populations should translate to significant public health impacts, with > 30% reductions in both parasite prevalence and clinical incidence predicted across a wide range of transmission settings and across a range of potential values for the excess mortality.

While theoretically, reductions in entomological endpoints should lead to reductions in epidemiological endpoints, this relationship is not always clear empirically. However, it is worth noting that the observed reductions in mosquito catch and EIR are similar to or greater than those observed to date in cluster-randomized studies of other VCTs. For example, a large cluster-randomized trial of ITNs in Western Kenya [24] showed a 58.5% reduction in *Anopheles gambiae* and 90% reduction in *Anopheles funestus* captured using pyrethrum spray collection*.* This same trial resulted in a reduction in malaria incidence and prevalence in young children of 60% and 19% respectively [25, 26]. However, extrapolation from entomological endpoints alone cannot be made for ITNs since their efficacy will represent both direct and indirect protection. Whilst there are no cluster RCTs of IRS alone [27], in recent years the impact of LLINs and IRS on mosquito populations has been assessed in two large cluster-randomized trials comparing the benefits of combining IRS and LLINs. In the first in the Gambia (in which IRS did not show any additional epidemiological benefit in addition to LLINs), mosquito counts were 33% lower in the LLIN + IRS group compared to the LLIN-only group, but this difference was not statistically significant. In this study, the EIR was low and not statistically different between intervention arms. In the second trial in Tanzania mosquito counts were reduced by 29% and EIR by 87% in the LLIN + IRS arm compared to the LLIN-only arm, although these differences were of marginal statistical significance. However, this translated to a 50% reduction in parasite prevalence in the LLIN + IRS arm compared to the LLIN-only arm. The projected epidemiological impact of ATSBs based on the observed entomological endpoints therefore appears plausible but requires confirmation in epidemiological randomized trials.

One of the key parameters determining the likely efficacy of ATSBs is the excess mortality, which, given the lethality of the toxin, is primarily determined by the rate at which mosquitoes feed on the bait. From the observed reduction in mosquito catch numbers, this excess mortality is estimated to be in the range ~ 0.07–0.11/day, effectively at least doubling the natural death rate of *Anopheles* mosquitoes. These estimates are notably lower than the estimates of the bait feeding rate obtained by Marshall et al*.* [8] (0.40/day) and the values estimated here in the one-day staining experiments using dyed, non-toxic bait (0.28–0.38/day in the period of ATSB use) although they are more consistent with the bait feeding rate in the control arm of the study by Marshall et al*.* (0.15/day for female mosquitoes).

There are a number of possible explanations for this discrepancy. Firstly, the base mosquito mortality may be higher than the natural mortality rate due to the presence of other vector control interventions, as noted in Sect. 3.2. This can lead to an under-estimation of the ATSB mortality rate, as shown in Fig. 2b, where the extrapolated ATSB mortality rate increases if the base mortality rate is increased. In addition, short-term experiments may give rise to higher values than those observed over longer time periods due to variations in the bait-feeding rate between mosquitoes—if some mosquitoes are more disposed to feed on the bait than others, these will be killed early on and over time the bait-feeding rate may decline.

Another possibility is that migration of mosquitoes from areas unaffected by the ATSBs acts to mitigate the population reduction and produce a lower apparent increase in the death rate. The villages in which the data reported here was gathered are located in the flood plain of the Niger river with rice paddies and other breeding sites nearby. In contrast, the previous small-scale village studies in central Mali [8] were carried out at the end of the rainy season without significant nearby breeding sites and this difference may account for the higher apparent excess mortality. Thus, whilst the one-day staining experiments can continue to provide useful information, particularly on the variation in the bait feeding rate between different ecological locations [11, 12, 14, 28], it will be important to continue to collect entomological outcomes in future RCTs of this new intervention.

The bait feeding rate estimated here from the one-day staining experiments was found to be negatively correlated with the rainfall level, suggesting that the ATSB efficacy may vary seasonally. This seasonal effect may be due to greater availability of natural sugar sources such as flowers and fruit inside villages during the wet season, providing alternative attractant sugar sources for both male and female mosquitoes. Alternatively, the observed feeding rates of mosquitoes inside the villages may be lowered by large numbers of already sugar fed mosquitoes invading the villages from nearby breeding sites. If this is the case, it suggests that the overall efficacy of ATSBs can be expected to vary significantly between ecological and/or geographical settings, with locations where natural sugar sources are more abundant showing reduced efficacy. In this context, the impact of invasive plants flowering during the dry season also needs to be considered [28]. Previous studies of ATSB in arid environments with varying levels of natural sugar availability suggest that natural sugar sources have only a delaying effect on ATSB efficacy [13]. However, further data are needed from the range of malaria-endemic environments to confirm this.

There are a number of limitations to this study. Firstly, the projected impact of ATSBs made here are based on the results of a single field study in a single ecological zone. These results should, therefore, be interpreted as indicative rather than predictive. Secondly, as noted above, the excess mortality can be expected to vary between different ecological environments and at different times of the year, depending on the availability of alternative sugar sources. Thus, whilst a major impact on both malaria and clinical incidence is predicted across a range of potential excess mortality values, if the feeding rate on the bait is very low then a sharp reduction in efficacy would be expected. Thirdly, trials have not yet been carried out to assess the effect of ATSBs on epidemiological outcomes in the field. Evidence from past VCT trials has demonstrated that it can be difficult to extrapolate epidemiological outcomes based on entomological outcomes alone given that many other factors could vary between settings. Finally, the large impact of ATSBs on mosquito catch numbers observed in the study in Mali, if replicated elsewhere, could be expected to exert a strong selective pressure. Adaptation of mosquito behaviour in response to the presence of ATSBs could reduce efficacy in the longer-term, making it difficult to predict the sustainability of this new tool, although it may be possible to mitigate this through the use of multiple toxins.

## Conclusions

The results from the first cluster-randomized study of ATSBs suggest that this new tool could provide a promising addition to existing VCTs and result in significant reductions in malaria burden across a range of malaria-endemic settings. These estimates can help to inform the design of future cluster randomized field studies in different ecological settings and/or incorporating epidemiological outcomes that will be required to confirm the efficacy of ATSBs as a public health intervention.

## Supplementary Information

**Additional file 1.** Additional information.

## Data Availability

We will make available, on request and via official databases where possible, all field data contributing to the work presented here along with all code and output data relating to data extrapolation.

## Abbreviations

ATSB: Attractive targeted sugar bait (sometimes referred to as attractive toxic sugar bait)
CDC: Centre for Disease Control
EIR: Entomological inoculation rate
HLC: Human landing catch
IRS: Interior residual spraying
ITN: Insecticide-treated net
LLIN: Long-lasting insecticide-treated net
PSC: Pyrethroid spray catch
RCT: Randomized clinical trial
SAS: Statistical Analysis System
VCT: Vector control tool
