# Supplementary material for: Estimating the potential impact of Attractive Targeted Sugar Baits (ATSBs) as a new vector control tool for Plasmodium falciparum malaria
Source: Malar J. 2021 Mar 17;20:151. doi: 10.1186/s12936-021-03684-4 (PMC7968277; doi:10.1186/s12936-021-03684-4)
Supplement: Supplementary file 1 — Additional file 1. Additional information. [file 12936_2021_3684_MOESM1_ESM.docx]

Supplementary Information

**1. Mathematical model of *Plasmodium falciparum* malaria transmission**

The model described here is an extension of a model published in 2010 by Griffin et al. (1) and subsequently re-fitted to capture the relationship between exposure and clinical disease (2). It has been further extended to capture the mosquito dynamics as described in White et al. (3). In this work we used the deterministic version of the model. A summary of the model is given in the main text whilst the full mathematical details are described below.

**1.1 Infection categories**

Figure S1 shows the model in diagram form, encompassing the six human infection categories S, T, D, A, U and P and transitions between them due to infection, passage between different stages of infection, and recovery.

Susceptible individuals (S) are infected at a rate *Λ* which depends on the entomological inoculation rate (EIR). After a fixed 12-day latent period (denoted by the circle in the centre of the diagram), they either develop symptoms (with probability *Φ*) or proceed with asymptomatic infection (state A). The probability of developing symptoms, *Φ*, is determined by an individual’s level of exposure-driven immunity (see Section 0) which changes through the course of their lifetime. Those developing disease are either successfully treated (entering state T) or fail treatment or are untreated (entering state D). Those that are successfully treated subsequently enter a period of prophylaxis (P) with the duration of this state depending on the drug used. Following this, individuals return to the susceptible state S. Those that fail treatment or are not treated are assumed to self-resolve and become asymptomatic (entering state A). As only a small proportion of cases are fatal, we do not explicitly model deaths in the transmission cycle. Individuals with asymptomatic infection subsequently move to a sub-patent state (U) which allows the model to capture onward infection from individuals with low parasite densities. Super-infection can occur from states A and U. All states are also stratified by the rate at which people are bitten by mosquitoes to capture heterogeneity in exposure.

The differential equations for the human transmission model are as follows:

$\frac{\partial S}{\partial t}+\frac{\partial S}{\partial a}=- \Lambda S+\frac{P}{d_{P}}+\frac{U}{d_{U}}$ **(S1a)**

$\frac{\partial T}{\partial t}+\frac{\partial T}{\partial a}=\phi f_{T} \Lambda\left( S+A+U \right)-\frac{T}{d_{T}}$ **(S1b)**

$\frac{\partial D}{\partial t}+\frac{\partial D}{\partial a}=\phi\left( 1-f_{T} \right) \Lambda\left( S+A+U \right)-\frac{D}{d_{D}}$ **(S1c)**

$\frac{\partial A}{\partial t}+\frac{\partial A}{\partial a}=\left( 1-\phi\right) \Lambda\left( S+U \right)+\frac{D}{d_{D}}-\phi\Lambda A-\frac{A}{d_{A}}$ **(S1d)**

$\frac{\partial U}{\partial t}+\frac{\partial U}{\partial a}=\frac{A}{d_{A}}-\frac{U}{d_{U}}- \Lambda U$ **(S1e)**

$\frac{\partial P}{\partial t}+\frac{\partial P}{\partial a}=\frac{T}{d_{T}}-\frac{P}{d_{P}}$ **(S1f)**

where $t$ denotes time and $a$ denotes age. Here $f_{T}$ is the proportion of clinical cases receiving effective treatment and $d_{T},d_{D},d_{A},d_{P},d_{U}$ denote the mean duration of states T, D, A, P and U respectively.

Variation in exposure to mosquitoes is included to capture both increased biting with age and other sources of heterogeneity in exposure (for example, geographic variation). Each individual has a relative biting rate $\zeta$ which is drawn from a Log-Normal distribution with parameters $-\sigma^{2}/2$ and $\sigma$ , such that $\zeta$ has a mean of 1. If $\varepsilon_{0}$ is the mean EIR in adults (see Section 0), then the EIR (*ε*) and force of infection ($\Lambda$) at age $a$ are given by the equation:

$\Lambda=b\varepsilon=b\varepsilon_{0}\zeta\left( 1-\rho exp\left( -\frac{a}{a_{0}} \right) \right)$ **(S2)**

where $b$ is the probability of infection if bitten by an infectious mosquito, and $\rho$ and $a_{0}$ determine how the rate of being bitten changes with age.

**1.2 Acquisition and decay in naturally acquired immunity**

To capture the natural acquisition of immunity to malaria disease and infection, we define three empirical functions that apply at different stages of human infection.

- Pre-erythrocytic immunity, $I_{B}$ : this is assumed to reduce the probability of infection if bitten by an infectious mosquito;
- Acquired and maternal clinical immunity, $I_{CA}$ and $I_{CM}$ respectively: the effect of blood stage immunity in reducing the probability of developing clinical symptoms;
- Detection immunity, $I_{D}$ : the effect of blood stage immunity in reducing the detectability of an infection and onward transmission to mosquitoes.

Each of the four acquired immunity functions are assumed to change with exposure as follows, starting from zero at birth:

$\frac{\partial I_{B}}{\partial t}+\frac{\partial I_{B}}{\partial a}=\frac{\varepsilon}{\varepsilon u_{B}+1}-\frac{I_{B}}{d_{B}}$ **(S3a)**

$\frac{\partial I_{CA}}{\partial t}+\frac{\partial I_{CA}}{\partial a}=\frac{\Lambda}{\Lambda u_{C}+1}-\frac{I_{CA}}{d_{C}}$ **(S3b)**

$\frac{\partial I_{VA}}{\partial t}+\frac{\partial I_{VA}}{\partial a}=\frac{\Lambda}{\Lambda u_{V}+1}-\frac{I_{VA}}{d_{V}}$ **(S3c)**

$\frac{\partial I_{D}}{\partial t}+\frac{\partial I_{D}}{\partial a}=\frac{\Lambda}{\Lambda u_{D}+1}-\frac{I_{D}}{d_{ID}}$ **(S3d)**

In each equation, $u_{K},k=B,CA,VA,D$ limits the rate at which immunity can be boosted at high exposure and each $d_{K},k=B,C,V,D$ determines the duration of immunity.

The immunity functions are transformed by Hill functions to give the probabilities of each transition affected by immunity. The probability of infection decreases from $b_{0}$ with no immunity to a minimum of $b_{0}b_{1}$, and is given by the following formula where $I_{B0}$ and $\kappa_{B}$ are scale and shape parameters respectively:

$b=b_{0}\left( b_{1}+\frac{1-b_{1}}{1+\left( I_{B}/I_{B0} \right)^{\kappa_{B}}} \right)$ **(S4)**

At birth, maternal clinical immunity $I_{CM}$ is $P_{M}$ multiplied by the acquired immunity of a 20-year-old, then decays at rate $1/d_{M}$. The probability of clinical disease is $\phi_{0}$ with no immunity, has a minimum of $\phi_{0}\phi_{1}$, and is given by the following formula where $I_{C0}$ and $\kappa_{C}$are scale and shape parameters respectively:

$\phi=\phi_{0}\left( \phi_{1}+\frac{1-\phi_{1}}{1+\left( (I_{CA}+I_{CM})/I_{C0} \right)^{\kappa_{C}}} \right)$ **(S5)**

The probability of detecting an asymptomatic infection by microscopy is:

$q=d_{1}+\frac{(1-d_{1})}{\left( 1+(I_{D}/I_{D0})^{\kappa_{D}}f_{D} \right)}$ **(S6)**

where $d_{1}$ is the minimum probability, $I_{D0}$ and $\kappa_{D}$are scale and shape parameters, and $f_{D}$ is given by $f_{D}=1-(1-f_{D0})/\left( 1+(a/a_{D})^{\gamma_{D}} \right)$ at age $a$, with parameters $f_{D0}$, $a_{D}$ and $\gamma_{D}$.

This reduced detectability is assumed to result from a lower parasite density, which also reduces the probability of infecting a mosquito if bitten. In states D and U, infectiousness is $c_{D}$ and $c_{U}$respectively. Following treatment, infectivity is $c_{T}$. In state A, infectiousness is $c_{U}+(c_{D}-c_{U})q^{\gamma_{I}}$where $q$ is the probability of detection of parasites. In states A and D there is a lag of $t_{L}$ between entering the infectious state and becoming onwardly infectious, to represent the delay between acquiring parasites and developing gametocytes.

The estimates of the parameters for the human part of the transmission model, and their 95% credible intervals where fitted, are given in Table S1. Values are taken from the datasets used in Griffin et al. 2014 [2].

**1.3 Mosquito dynamics**

The full lifecycle of the mosquito is modelled in a compartmental formulation [3]. Female adult mosquitoes (M) lay eggs at rate *β* which then develop through the early and late larval stages (E and L) to the pupal stage (P_L_). The death rate of the larval stages is increased by a density-dependent factor. Pupae emerge as adult mosquitoes, with 50% being female, which are susceptible to infection (S_M_). The differential equations are:

$\frac{dE}{dt}=\beta M-\mu_{E}\left( 1+\frac{E+L}{K} \right)E-\frac{E}{d_{EL}}$ **(S7a)**

$\frac{dL}{dt}=\frac{E}{d_{EL}}-\mu_{L}\left( 1+\gamma\frac{E+L}{K} \right)L-\frac{L}{d_{L}}$ **(S7b)**

$\frac{dP_{L}}{dt}=\frac{L}{d_{L}}-\mu_{P}P_{L}-\frac{P_{L}}{d_{PL}}$ **(S7c)**

$\frac{dS_{M}}{dt}=\frac{P_{L}}{{2d}_{PL}}-\mu_{M}S_{M}$ **(S7d)**

where each $\mu$ is a death rate, each $d$ is the duration in a state, and $\gamma$ modifies the effect of density dependence on late stage relative to early stage larvae. $K$ is the time-varying carrying capacity, the ability of the environment to support mosquito larvae, which determines the mosquito density and hence the baseline transmission intensity in the absence of interventions. This carrying capacity is either held constant (for non-seasonal runs) or is varied as proportional to rainfall (for seasonal runs).

Adult mosquitoes become infected at a rate which depends on the infectiousness of the human population, which in turn depends on the infection states at time $t_{l}$ previously, where $t_{l}$ represents the time lag in humans from asexual parasites to onward infectiousness. The force of infection on mosquitoes is the sum of the contributions from the different human infection states:

$\Lambda_{M}=\frac{\alpha}{\omega}\int_{0}^{\infty} \int_{0}^{\infty} \zeta\psi(a)\left( c_{D}D(\zeta,a,t-t_{l})+c_{T}T(\zeta,a,t-t_{l})+c_{A}A(\zeta,a,t-t_{l})+c_{U}U(\zeta,a,t-t_{l}) \right)dad\zeta$ **(S8)**

The biting rate on humans is $\alpha=Q_{0}/\delta$, where $\delta$ is the mean time between feeds and *Q_0_* is the proportion of bites that are on humans (anthropophagy). $\omega=\int_{0}^{\infty} \psi(a)g(a)da$ is a normalizing constant for the biting rate over ages, where $g(a)$ is the cross-sectional human age distribution.

Once infected, mosquitoes pass through a latent period (E_M_) of fixed length $\tau_{M}$ and then they become infectious to humans (*I_M_*). They are assumed to remain infectious until they die. The infection process in the mosquito population is as follows:

$\frac{dS_{M}}{dt}=\frac{P_{L}}{{2d}_{PL}}-\Lambda_{M}S_{M}-\mu_{M}S_{M}$ **(S9a)**

$\frac{dE_{M}}{dt}=\Lambda_{M}S_{M}-\Lambda_{M}\left( t-\tau_{M} \right)S_{M}\left( t-\tau_{M} \right)P_{M}-\mu_{M}E_{M}$ **(S9b)**

$\frac{dE_{M}}{dt}=\Lambda_{M}(t-\tau_{M})S_{M}(t-\tau_{M})P_{M}-\mu_{M}I_{M}$ **(S9c)**

where $P_{M}=e^{-\mu_{M}\tau_{M}}$ is the probability that a mosquito survives the extrinsic incubation period. The mean EIR for adult humans is then given by $\varepsilon_{0}=\frac{\alpha}{\omega}I_{M}$ . The parameter values for the mosquito population model, which are drawn from the datasets used in White *et al* [3] are given in Table S2.

**1.4 Prevalence and incidence credible intervals**

The credible intervals shown for modelled prevalence and incidence data in the main body of the paper were generated using sets of parameter values taken from a distribution of 1000 parameter value sets drawn from the Markov chain Monte Carlo derivation of the best-fit estimates shown in Tables S1 and S2. The 1000 parameter value sets were ordered according to the average of the steady-state prevalence or incidence values across the EIR range shown in Fig. 4A-B, and the 25^th^ and 975^th^ sets taken as the ones representing the lower and upper bounds of the 95% credible intervals for prevalence or incidence.

Note that since the relationship between prevalence and incidence varies depending on the parameter values, the parameter sets used for the prevalence credible interval are different to those used for the incidence credible intervals. The chosen parameter value sets were used in the model to generate additional prevalence and incidence data which was used as the upper and lower bounds of the credible intervals shown in Figs. 4A-B, 5A-B, 6A-B and 7A-B.

**2. Parameter Estimates for Longitudinal Models to Estimate Differences in Mosquito Density and EIR**

In the main paper we estimated the impact of the ATSBs on two entomological endpoints – mosquito catch numbers and EIR – by fitting a non-linear model to the count data. The estimates of the parameters fitted to mosquito density are given in Table S3 and those for fitting to EIR in Table S4.

**Supplementary Information References**

1. Griffin JT, Hollingsworth TD, Okell LC, Churcher TS, White MT, Hinsley W, et al. Reducing Plasmodium falciparum malaria transmission in Africa: A model-based evaluation of intervention strategies. PLoS Med. 2010;7:e1000324.

2. Griffin JT, Ferguson NM, Ghani AC. Estimates of the changing age-burden of Plasmodium falciparum malaria disease in sub-Saharan Africa. Nat Commun. 2014;5:3136.

3. White MT, Griffin JT, Churcher TS, Ferguson NM, Basáñez MG, Ghani AC. Modelling the impact of vector control interventions on Anopheles gambiae population dynamics. Parasites and Vectors. 2011;4:153.


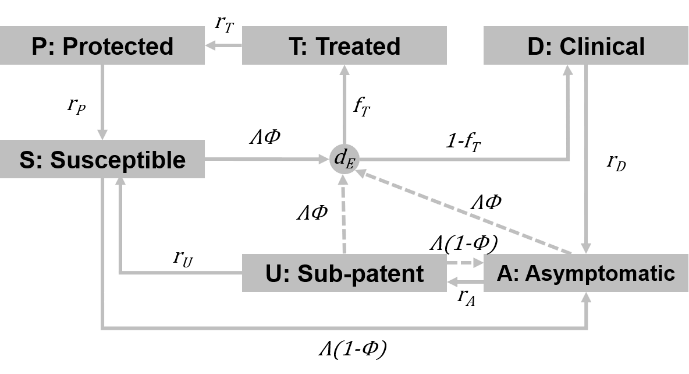


**Figure S1.** Diagram of human component of transmission model, showing infection categories *S, T, D, A, U, P*, latent period *d_E_*, rates of transfer between categories ($r_{T},r_{D},r_{A},r_{P},r_{U}$ equal to inverses of mean durations $d_{T},d_{D},d_{A},d_{P},d_{U}$), treatment probability *f_T_*, probability of clinical infection *Φ*, and force of infection *Λ*.

| **Parameter description** | **Symbol** | **Estimate (95% credible interval)** |
| --- | --- | --- |
| **Human infection durations (all in days)** | | |
| Latent period | $d_{E}$ | 12 (fixed) |
| Patent infection | $d_{A}$ | 195 (fixed) |
| Clinical disease (treated) | $d_{T}$ | 5 (fixed) |
| Clinical disease (untreated) | $d_{D}$ | 5 (fixed) |
| Sub-patent infection | $d_{U}$ | 110 (70,146) |
| Prophylaxis following treatment | $d_{P}$ | 20 (fixed) |
| **Infectiousness to mosquitoes** | | |
| Lag from parasites to infectious gametocytes | $t_{L}$ | 12.5 days (fixed) |
| With untreated disease or no immunity | $c_{D}$ | 0.068 day^-1^ (0.039, 0.122) |
| Treated disease | $c_{T}$ | 0.022 (fixed) |
| Sub-patent infection | $c_{U}$ | 0.0062 day^-1^ (0.00056, 0.018) |
| Parameter for infectiousness of asymptomatic state | $\gamma_{I}$ | 1.82 (0.603, 8.54) |
| **Age and heterogeneity** | | |
| Age-dependent biting parameter | $\rho$ | 0.85 (fixed) |
| Age-dependent biting parameter | $a_{0}$ | 8 years (fixed) |
| Variance of log of heterogeneity in biting rates | $\sigma^{2}$ | 1.67 (fixed) |
| **Immunity reducing probability of detection** | | |
| Probability with maximum immunity | $d_{1}$ | 0.161 (0.089, 0.24) |
| Inverse of decay rate | $d_{ID}$ | 10 years (fixed) |
| Scale parameter | $I_{D0}$ | 1.58 (0.22, 6.18) |
| Shape parameter | $\kappa_{D}$ | 0.477 (0.280, 0.826) |
| Duration in which immunity is not boosted | $u_{D}$ | 9.45 days (3.57, 19.2) |
| Scale parameter relating age to immunity | $a_{D}$ | 21.9 years (19.2, 25.3) |
| Parameter relating age to immunity | $f_{D0}$ | 0.0071 (0.00035, 0.0259) |
| Shape parameter relating age to immunity | $\gamma_{D}$ | 4.81 (3.79, 6.26) |
| **Immunity reducing probability of infection** | | |
| Probability with no immunity | $b_{0}$ | 0.590 (0.389, 0.845) |
| Maximum relative reduction | $b_{1}$ | 0.5 (fixed) |
| Inverse of decay rate | $d_{B}$ | 10 years (fixed) |
| Scale parameter | $I_{B0}$ | 43.9 (20.1, 120) |
| Shape parameter | $\kappa_{B}$ | 2.16 (1.22, 2.93) |
| Duration in which immunity is not boosted | $u_{B}$ | 7.20 days (2.63, 15.0) |
| **Immunity reducing probability of clinical disease** | | |
| Probability with no immunity | $\phi_{0}$ | 0.792 (0.548, 0.961) |
| Maximum relative reduction | $\phi_{1}$ | 0.00074 (0.00005, 0.0025) |
| Inverse of decay rate | $d_{C}$ | 30 years (fixed) |
| Scale parameter | $I_{C0}$ | 18.0 (11.9, 26.7) |
| Shape parameter | $\kappa_{C}$ | 2.37 (1.99, 2.86) |
| Duration in which immunity is not boosted | $u_{C}$ | 6.06 days (2.82, 11.1) |
| New-born immunity relative to mother’s | $P_{M}$ | 0.774 (0.536, 0.981) |
| Inverse of decay rate of maternal immunity | $d_{M}$ | 67.7 days (59.0, 79.4) |

**Table S1.** Parameters for the baseline human transmission model; taken from parameter sets used in Griffin *et al* [2]

| **Parameter description** | **Symbol** | **Estimate (95% credible interval)** |
| --- | --- | --- |
| Daily mortality of adult mosquitoes | $\mu_{M}$ | 0.096 day^-1^ (0.087, 0.110) |
| Per capita daily mortality rate of early stage larvae (low density) | $\mu_{E}$ | 0.034 day^-1^ (0.024, 0.044) |
| Per capita daily mortality rate of early stage larvae (low density) | $\mu_{L}$ | 0.035 day^-1^ (0.025, 0.044) |
| Per capita daily mortality rate of pupae | $\mu_{P}$ | 0.25 day^-1^ (0.18, 0.32) |
| Mean time between bites in humans | $\delta$ | 3 days (fixed) |
| Development time of early stage larvae | $d_{EL}$ | 6.64 days (4.82, 8.53) |
| Development time of late stage larvae | $d_{L}$ | 3.72 days (2.03, 5.61) |
| Development time of pupae | $d_{PL}$ | 0.64 days (0.07, 1.47) |
| Number of eggs laid per oviposition per mosquito | *ε_max_* | 73.7 (40.15, 89.2) |
| Relative effect of density dependence on late instars relative to early instars | $\gamma$ | 13.25 (9.82, 17.51) |
| Extrinsic incubation period | $\tau_{M}$ | 10 days (fixed) |

**Table S2.** Parameters for the mosquito population model; taken from parameter sets used in White *et al* [3]

| **Parameter** | **Estimate** | **Standard Error** | **P value** |
| --- | --- | --- | --- |
| **Pre-ATSB (2016)** |  |  |  |
| *a_M_* | 193.34 | 31.6360 | <.0001 |
| *b_M_* | 1.5375 | 0.00608 | <.0001 |
| *c_M_* | 2.5036 | 0.02016 | <.0001 |
| *d_M_* | 321.73 | 52.5766 | <.0001 |
| *β_M_* | -0.02668 | 0.2307 | 0.9097 |
| $\sigma_{M,r}^{2}$ | 0.1855 | 0.07020 | 0.0203 |
| **Post-ATSB (2017)** |  |  |  |
| *a_M_* | 161.10 | 25.1864 | <.0001 |
| *b_M_* | 1.5882 | 0.01279 | <.0001 |
| *c_M_* | 3.0325 | 0.03811 | <.0001 |
| *d_M_* | 245.69 | 38.3350 | <.0001 |
| *β_M_* | 0.8478 | 0.2204 | 0.0020 |
| $\sigma_{M,r}^{2}$ | 0.1690 | 0.06408 | 0.0205 |

**Table S3.** Results from the model fitting of the mosquito density data. The top panel shows the results in 2016 in which randomisation was conducted but the villages randomised to treatment arm received no intervention whilst the bottom panel shows the results following the application of intervention (ATSB) in the villages randomised to the treatment arm.

| **Parameter** | **Estimate** | **Standard Error** | **P value** |
| --- | --- | --- | --- |
| **Outdoors** |  |  |  |
| *a_EIR_* | 1.6220 | 0.1471 | <.0001 |
| *b_EIR_* | 1.1782 | 0.06389 | <.0001 |
| *c_EIR_* | 3.0353 | 0.2862 | <.0001 |
| *d_EIR_* | 1.5192 | 0.2465 | <.0001 |
| *R_EIR_* | 0.9281 | 0.08100 | <.0001 |
| $\sigma_{EIR,r}^{2}$ | 0.3328 | 0.1544 | 0.0505 |
| $\sigma_{EIR,e}^{2}$ | 0.5219 | 0.08054 | <.0001 |
| **Indoors** |  |  |  |
| *a_EIR_* | 1.6095 | 0.1191 | <.0001 |
| *b_EIR_* | 1.1877 | 0.05130 | <.0001 |
| *c_EIR_* | 3.0623 | 0.2292 | <.0001 |
| *d_EIR_* | 1.6050 | 0.2125 | <.0001 |
| *R_EIR_* | 0.8903 | 0.06617 | <.0001 |
| $\sigma_{EIR,r}^{2}$ | 0.2586 | 0.1163 | 0.0445 |
| $\sigma_{EIR,e}^{2}$ | 0.3386 | 0.05224 | <.0001 |

**Table S4.** Results from the model fitting of the EIR data in 2017. The top panel shows the results from mosquito count data obtained from Human Landing Catch method conducted outdoors whilst the bottom panel shows results from mosquito count data obtained from Human Landing Catch method conducted indoors.
